# Restoring Natural Killer Cell Immunity against Multiple Myeloma in the Era of New Drugs

**DOI:** 10.3389/fimmu.2017.01444

**Published:** 2017-11-07

**Authors:** Gianfranco Pittari, Luca Vago, Moreno Festuccia, Chiara Bonini, Deena Mudawi, Luisa Giaccone, Benedetto Bruno

**Affiliations:** ^1^Department of Medical Oncology, National Center for Cancer Care and Research, HMC, Doha, Qatar; ^2^Unit of Immunogenetics, Leukemia Genomics and Immunobiology, IRCCS San Raffaele Scientific Institute, Milano, Italy; ^3^Hematology and Bone Marrow Transplantation Unit, IRCCS San Raffaele Scientific Institute, Milano, Italy; ^4^Department of Oncology/Hematology, A.O.U. Città della Salute e della Scienza di Torino, Presidio Molinette, Torino, Italy; ^5^Department of Molecular Biotechnology and Health Sciences, University of Torino, Torino, Italy; ^6^Experimental Hematology Unit, Division of Immunology, Transplantation and Infectious Diseases, IRCCS San Raffaele Scientific Institute, Milano, Italy; ^7^Vita-Salute San Raffaele University, Milano, Italy

**Keywords:** multiple myeloma, immunotherapy, natural killer cells, killer immunoglobulin-like receptors, cytokines, immune checkpoint inhibition, daratumumab, elotuzumab, IDO inhibitors, chimeric antigen receptor

## Abstract

Transformed plasma cells in multiple myeloma (MM) are susceptible to natural killer (NK) cell-mediated killing *via* engagement of tumor ligands for NK activating receptors or “missing-self” recognition. Similar to other cancers, MM targets may elude NK cell immunosurveillance by reprogramming tumor microenvironment and editing cell surface antigen repertoire. Along disease continuum, these effects collectively result in a progressive decline of NK cell immunity, a phenomenon increasingly recognized as a critical determinant of MM progression. In recent years, unprecedented efforts in drug development and experimental research have brought about emergence of novel therapeutic interventions with the potential to override MM-induced NK cell immunosuppression. These NK-cell enhancing treatment strategies may be identified in two major groups: (1) immunomodulatory biologics and small molecules, namely, immune checkpoint inhibitors, therapeutic antibodies, lenalidomide, and indoleamine 2,3-dioxygenase inhibitors and (2) NK cell therapy, namely, adoptive transfer of unmanipulated and chimeric antigen receptor-engineered NK cells. Here, we summarize the mechanisms responsible for NK cell functional suppression in the context of cancer and, specifically, myeloma. Subsequently, contemporary strategies potentially able to reverse NK dysfunction in MM are discussed.

## Introduction

Multiple myeloma (MM) is a B-cell malignancy characterized by an abnormal growth of malignant plasma cells which derive from a post-germinal B-cell of the lymphoid cell lineage. The treatment paradigm for MM has undergone a dramatic evolution in the past decade given a considerable improvement in the understanding of disease pathogenesis. Despite the development of novel therapeutic agents such as proteasome inhibitors—bortezomib, carlfizomib—and immunomodulatory drugs—lenalidomide, pomalidomide—which target not only MM cells but also their interplay with the microenvironment, MM remains an incurable disease and the prognosis of patients with relapsed/refractory MM remains very poor. A number of factors concur to make MM a hard-to-treat hematologic malignancy. Drug resistance remains a major concern. MM is a highly heterogeneous disease with pathogenic processes that may greatly differ among newly diagnosed patients and others that may arise during the disease course. In recent years, several studies have focused on mechanisms of drug resistance even though many are not yet completely understood. It is widely assumed that cytogenetic and epigenetic abnormalities, deregulated signaling pathways, the MM bone marrow (BM) microenvironment, and the MM stem cell itself are all elements which play significant roles in drug resistance. Deletion 17p13 is one of the most relevant chromosomal abnormalities present in approximately 10–15% of newly diagnosed patients and observed more frequently in refractory-relapsed patients. It has been associated with resistance to new agents such as bortezomib and lenalidomide (1, 2). Aberrant drug transport processes and anti-apoptosis mechanisms have also been correlated with drug resistance (3, 4). Moreover, a pivotal role is played by the intense cell–cell crosstalk between the BM microenvironment and MM cells and their interplay with the extracellular matrix (5). All the abovementioned mechanisms make MM very challenging to eradicate with single-agent or combination modalities. Thus, an urgent need exists for new therapeutic strategies to overcome resistance to current therapies. MM is also characterized by a gradual and progressive immune dysregulation with impairs functions of B and T cell immunity, natural killer (NK) cells, and antigen-presenting/dendritic cells that allow malignant plasma cells to escape immunosurveillance. The combination of an “immunosuppressive” microenvironment and clonal evolution activate signaling pathways that invariably promote disease survival and progression. Several immunotherapies have recently been proposed and, among others, they have included monoclonal antibodies, antibody–drug conjugates, chimeric antigen receptor T cell therapy (CAR-T cells), tumor vaccines, and immune checkpoint inhibitors. This review provides an overview of the biological functions and potential clinical role of NK cells as a form of immunotherapy that may improve MM clinical outcomes.

## Physiology of NK Cells and Their Receptors

### Missing-Self Recognition and Inhibitory NK Cell Receptors

In the early 1970s, immune effectors isolated from mice and humans were found to display *in vitro* antitumor cytotoxicity without prior immunization by tumor antigens *in vivo* (6–9). These cells were functionally defined as *N-cells* or *NK cell*s and were believed to belong to the lymphoid lineage, but to be distinct from B and T cells (10–13). Mechanisms regulating NK cell-mediated target recognition and killing remained obscure for more than a decade after natural cytotoxicity was first described. In 1986, Karre et al. reported that resistance of mice lymphoma cells to NK cell-mediated rejection was dependent on major histocompatibility complex (MHC) class I antigen expression on cancer surface (14). This observation led to the assumption that NK cell would possess receptors able to transduce negative signals upon MHC class I engagement, thus sparing putative targets. Lack of MHC class I would instead trigger NK cell activation, a phenomenon known as *missing-self* recognition (15).

In humans, the NK cell inhibitory receptors able to recognize HLA class I are type I transmembrane structures belonging to the immunoglobulin (Ig) superfamily, known as killer immunoglobulin-like receptors (KIR). Inhibitory KIR share a long (L) cytoplasmic tail containing immunoreceptor tyrosine-based inhibitory motifs that can process signals through the recruitment and activation of the SH2-domain-containing tyrosine phosphatase 1 protein (16–20). Three inhibitory KIR engaging HLA class I ligand groups are critical regulators of NK cell function: KIR2DL1, specific for HLA-C2 group antigens (sharing Asn at position 77 and Lys at position 80 of the HLA-Cw heavy chain); KIR2DL2/3, specific for HLA-C1 group antigens (sharing Ser at position 77 and Asn at position 80 of the HLA-Cw heavy chain) (21, 22); and KIR3DL1, specific for the HLA-Bw4 epitope (located at position 77–83 of the heavy chain of certain HLA-B and HLA-A alleles) (23–25).

In the last two decades, multiple additional inhibitory NK cells receptors have been identified, leading to the currently accepted notion that NK cell effector function is dependent on the overall balance of signals transduced by multiple inhibitory and activating receptors recognizing cognate ligands on virally infected and cancer cells. Examples of non-KIR inhibitory NK receptors include the c-type lectin-like CD94/NKG2A (CD159a) heterodimer and ILT2 (LILRB1, CD85j), respectively, engaging HLA-E and various HLA class I antigens (26, 27); NKR-P1A (CD161) recognizing the lectin-like transcript 1 (28, 29); and the carcinoembryonic antigen-related cell adhesion molecule 1 (CD66a) recognizing the CD66 ligand (30–32).

### Activating NK Cell Receptors

Activating NK cell receptors are also described. Among them, NKG2D (CD314) has ligand specificity for a wide range of stress-induced cell surface ligands (NKG2D-L), including the MHC-related ligands MICA and MICB (33) and the human cytomegalovirus glycoprotein (UL16)-binding proteins ULBP1-6 (33, 34). Natural cytotoxicity receptors (NCRs) NKp46 (NCR1, CD335) (35, 36), NKp44 (NCR2, CD336) (37), and NKp30 (NCR3, CD337) (38) are potent activating receptors almost exclusively restricted to NK cells. Ligands for NCR are currently incompletely characterized. NKp46 and NKp44 are known to bind several viral hemagglutinins (39, 40), while NKp30 recognizes the HLA-B-associated transcript 3 (BAT3) (41) and B7-H6, a member of the B7 immunoreceptor family (42). CD94/NKG2C (CD159c) binds the non-classical HLA-E, similar to its inhibitory CD94/NKG2A counterpart (25). CD16 (FcγRIIIA) (43) is the low-affinity IgG receptor, strongly expressed on mature NK cells, mediating antibody-dependent cellular cytotoxicity (ADCC) (44). Other important activating receptors include the SLAM-related 2B4 (CD244) (45) engaging the pan-leukocyte surface antigen CD48 (46) and the adhesion molecule DNAM-1 (47) involved in recognition of PVR (CD155) and nectin-2 (CD112) (48).

## NK Cell Immunity Dysfunction in MM

### Tumor-Induced Microenvironment Transformation

Accumulating evidence indicates that microenvironment transformation may significantly impair NK cell effector function in MM (49). Plasma cells and T regulatory (T_reg_) cells from patients with MM secrete high levels of TGF-β (50, 51), a potent immunosuppressive cytokine known to downregulate multiple NK-activating receptors and to impair NK cytotoxicity (52–54). IL-10 and IL-6 are increased in MM (55–57) and independently act as powerful growth factors for malignant plasma cells (58, 59). IL-10 inhibits production of pro-inflammatory IFN-γ and TNF-α (60, 61) and promotes development of NK-resistant tumor phenotypes (62), although it may also enhance NK cytotoxicity in response to IL-15 exposure *in vitro* (63). IL-6 has been shown to impair NK cell activity in experimental models, human disease, and when administered to patients with advanced cancer (64–66). Altered levels of IFN-γ may also contribute to NK cell dysregulation in MM. In two studies, serum IFN-γ levels were found to be significantly lower in subjects with MM than in normal controls (55, 56), potentially affecting NK cell activity. Besides cytokines, other soluble factors are known to suppress NK-mediated antitumor capabilities. Prostaglandin E2 inhibits activating signals transduced by NCR, NKG2D, and CD16 (67) and has been shown to be actively produced in cultures of BM from patients with MM (68). Indoleamine 2,3-dioxygenase (IDO) promotes cancer cell immune escape through potent immunoregulatory effects on antigen-presenting cells *via* enzymatic degradation of l-tryptophan (69) (see IDO inhibitors). Della Chiesa et al. described that IDO-mediated immunosuppression also involves NK cells *via* l-kyreunine (Kyn), a l-tryptophan (Trp) degradation product impairing NKp46/NKG2D-specific lysis (70). Interestingly, interaction between CD28 on MM cells and CD80/86 stimulates IDO production by stromal dendritic cells (71), in agreement with the observation that CD28 expression on MM plasma cells is a marker correlating with poor disease outcome (72).

Additional microenvironmental factors may contribute to blunted NK cell cytotoxicity and cytokine production in MM. Among them, myeloid-derived suppressor cells (MDSCs) have been found to be expanded in MM (73, 74) and to directly contribute to downregulation of NK cell responsiveness *via* the NKp30-activating receptor (75), membrane-bound TGF-β (76), and TIGIT-mediated inhibitory signaling (77). Furthermore, reduced oxygenation described in MM BM (78, 79) may inhibit NK cell anti-myeloma responsiveness (80).

### Effect of Soluble Ligands on NK Cell-Mediated Immunity in MM

MICA and MICB (collectively named MIC) are stress-inducible NKG2D ligands frequently overexpressed in response to malignant transformation (81). When bound to tumor surface, they act as markers of “abnormal self” and may trigger NK cell cytotoxicity *via* NKG2D signaling. Conversely, cleavage of membrane-bound MIC is a strategy employed by MM and other tumors to evade NK cell immunosurveillance (82–85). In individuals with MIC^+^ tumors, soluble MIC (sMIC) ligands induce internalization of surface NKG2D (but also NCR and chemokine receptors) and substantial impairment of NK effector functions (86–88). In addition, sMIC has been shown to promote the accumulation of MDSC and macrophages with an immunosuppressive phenotype (89), potentially contributing to NK cell suppression. Not surprisingly, presence of sMIC is associated with poor cancer survival (90–92). In MM, shedding of MIC may result from exposure of MM cells to the genotoxic agents, doxorubicin and melphalan (93). Proteolytic cleavage by ADAMTS10 has been described to mediate this phenomenon, suggesting that the combination of metalloproteinase inhibitors with chemotherapy would exert a protective effect against escape of MM cells from NK-mediated recognition (93). Similar to NKG2D-L, NCR-specific soluble ligands may in some instances induce NK cell functional impairment. For example, circulating BAG6/BAT3 may inhibit NK cell cytotoxicity by inducing NKp30-specific hyporesponsiveness (94). Shedding of these ligands in the context of MM has not been investigated.

### Effect of Cell Contact on NK Cell-Mediated Immunity in MM

Derangement of NK cell effector functions may be further amplified by tumor ligand surface expression patterns favoring dominance of inhibitory NK signals. Ligands recognized by NK-activating receptors are often poorly expressed in cancer. Downregulation of membrane-bound NKG2D-L is common in multiple tumors, resulting in impaired NKG2D-dependent NK cell cytotoxicity (95–97) and unfavorable clinical outcomes (97). In the context of monoclonal gammopathy, expression of MICA is known to decrease upon transition from pre-cancerous monoclonal gammopathy of undetermined significance (MGUS) to MM (84). Of note, various pharmacological interventions may counter NKG2D-L downregulation in MM: vincristine, *via* p38 MAPK pathway activation (98); doxorubicin, melphalan, and bortezomib as a result of oxidative stress, DNA damage, and tumor senescence (99, 100) the heat shock protein-90 (HSP90) chaperone protein inhibitors 17-allylaminogeldanamycin and radicicol (101); and inhibition or degradation of bromodomain and extra-terminal proteins (102). Exposure to therapeutic agents with activity on MM has similarly been shown to induce upregulation of PVR (an activating ligand for DNAM-1) on malignant plasma cells (98, 100, 103). Besides NKG2D-L, surface expression of the B7-H6 ligand, engaging the NKp30 NCR, has been found to be downregulated in cell lines generated from multiple cancers, including MM, resulting in NKp30-dependent NK cell functional impairment (104).

Upregulation of tumor-bound HLA class I antigens is another mechanism of protection against NK cell immunosurveillance. Malignant plasma cells obtained from the BM of early-stage myeloma patients display low HLA class I expression potentially favoring NK-mediated killing (105). In contrast, high HLA class I levels are observed on plasma cells derived from pleural effusions of patients with advanced MM (105). HLA-E is a non-classical HLA class I antigen frequently upregulated on cancer cells, a phenomenon correlating with poor prognosis (106). In MM primary cells, high HLA-E expression results in restrained *in vitro* degranulation of NK cell subsets expressing the HLA-E-specific inhibitory NK receptor NKG2A (107).

Surface overexpression of ligands for inhibitory NK receptors is not restricted to HLA class I antigens. Notably, the CD200 glycoprotein is also commonly overexpressed on cancer surface, specifically in myeloid and lymphoid leukemias, where it is a marker of poor prognosis (108, 109). Leukemia blasts overexpressing CD200 escape NK-mediated immunosurveillance by dampening NK cell cytolytic capabilities and NKp44/NKp46 receptor expression (110), a phenomenon that can be reversed by CD200 blockade (111). CD200 is also frequently expressed in patients with MM, where it adversely affects clinical outcomes following stem cell transplantation (112).

### Numerical, Phenotypic, and Functional Characteristics of NK Cells in MM

**Figure 1:**
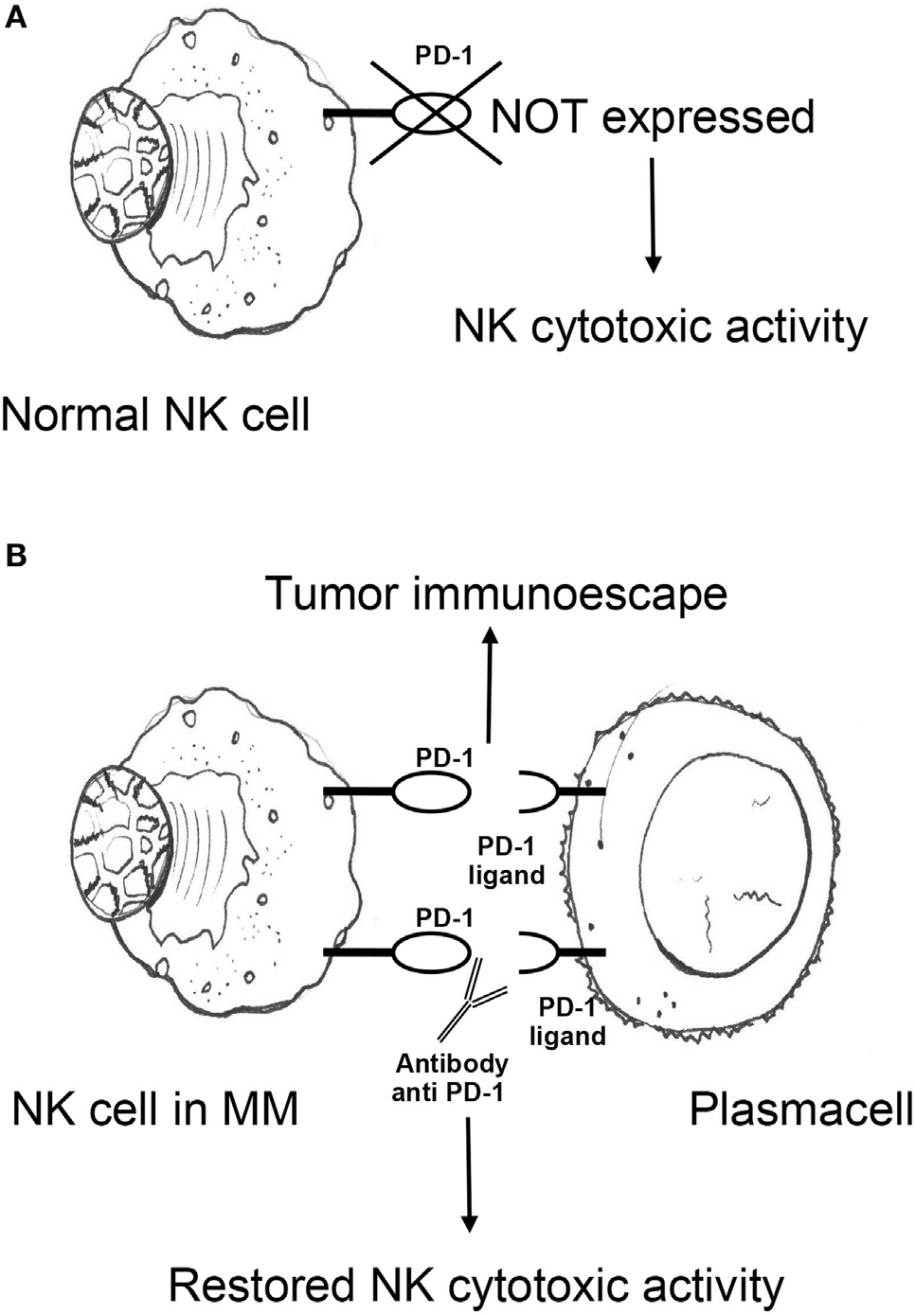
**(A)** Antitumor cytotoxic activity of NK cells in healthy individuals is not impaired by PD-1 expression. **(B)** NK cells from MM patients express PD-1, which promotes MM escape from NK cell-mediated immunosurveillance upon engagement with cognate ligand PD-L1 on plasma cells. PD-1/PD-L1 blocking monoclonal antibodies may potentiate NK cell effector functions against MM. NK, natural killer; PD-1, programmed death receptor-1; MM, multiple myeloma.

Multiple reports describe numerical, phenotypic, and functional NK cell alterations in MM. Subjects with MGUS and untreated, early-stage MM have been generally found to have higher (113–115) or similar (116–118) numbers of circulating and BM NK cells than healthy donors. Upregulation of CD57 and CD16 on NK cell surface is also observed (119, 120), suggesting the emergence of terminally differentiated subsets with high-cytotoxic potential. While these findings suggest efficient response to malignant clones subject to NK-mediated immunosurveillance, several lines of evidence favor the view that such early anti-MM effects are rather to be interpreted as a sign of immunological stress resulting in poor disease control. In fact, the effector function of expanded NK cells from MM subjects has been unexpectedly found to be similar to that of NK cells obtained from healthy donors (114), and NK cells obtained from untreated or previously treated MM patients show a lower increase in cytotoxicity to the K562 cell line in response to pre-incubation with IFN-γ (121). Moreover, NK cell effector functions positively correlate with presence of adverse prognostic factors, including anemia, low albumin, high β2-microglobulin, and renal failure (115), suggesting a “stressed” immunoresponse under the pressure of an aggressive clonal expansion (115). Notably, NK cells from patients with MM display an “exhausted” phenotype signature that includes downregulation of multiple activating receptors and upregulation of programmed death receptor-1 (PD-1). Surface expression of activating 2B4 is reduced in both PB (122) and BM (123) NK cells obtained from untreated subjects with MM, potentially preventing killing of plasma cells despite low HLA class I expression (105). NKG2D and NCR are also downregulated in MM, but preferentially in the BM (122, 123), supporting the concept that downregulation of certain activating NK cell receptors is both dependent on soluble ligands and direct cell–cell contact. Negative signaling from PD-1 is a well-established marker of exhaustion on T cells, but can also disrupt NK cell cytotoxicity and cytokine production (124). In MM, both expression of PD-1 on NK cells and of its ligand PD-L1 on plasma cells has been described (125, 126). PD-1/PD-L1 interactions may therefore promote NK cell functional exhaustion in MM, a phenomenon potentially reversible by checkpoint blockade inhibition (see Inhibitors of the PD-1/PD-L1 Pathway; Figure 1).

Natural killer cell-mediated immunity further deteriorates in advanced MM. Compared to MGUS and untreated MM, PB NK cell numbers are substantially reduced in advanced disease (113). Altered distribution of NK cell subsets in human BM may be likewise hypothesized based on studies in mice demonstrating selective decrease of KLRG1^−^ NK cells during MM progression (127). Evolving phenotype editing further promotes tumor escape from NK cell-mediated immunosurveillance. Furthermore, the activating receptor DNAM-1, expressed on NK cells from healthy donors and with MM in complete remission, is downregulated on NK cells from patient with active MM (128). This phenomenon is particularly relevant for late-stage cancer immune escape, as killing of malignant plasma cells is in certain circumstances critically dependent on DNAM-1 engagement of PVR and nectin-2 (128, 129). In line with these findings, NK cell activity in advanced MM is significantly impaired (130). Taken together, these data indicate that NK cell immunity alterations, already detectable in early myeloma, progress in a clinical stage-dependent manner and that immunotherapy modalities based on efficient NK cell effector function such as (i.e., mAbs) are likely to exert a more effective anti-myeloma effect when used in early-stage disease.

Factors promoting NK immunity suppression in MM are summarized in Table 1.

**Table 1:** Microenvironment alterations potentially promoting natural killer (NK) immunity suppression in multiple myeloma.

Factors	Function	Effect of TM	Impact on NK cell immunity	Reference
**Soluble**				
TGF-β	Anti-inflammatory cytokine	⇑	Reduced NK effector functions Downregulation of activating receptors	Castriconi et al. (52); Lee et al. (53); Mamessier et al. (54)
IL-10	Anti-inflammatory cytokine	⇑	Resistance to NK cytotoxicity Reduced NK cytokine production	Tsuruma et al. (62); Sharma et al. (55); Zheng et al. (56)
IL-6	Pro-inflammatory cytokine	⇑	Reduced NK effector functions	Bataille et al. (57); Scheid et al. (66)
IFN-γ	Pro-inflammatory cytokine	⇓	Reduced NK effector functions	Sharma et al. (55); Zheng et al. (56)
PGE2	Prostaglandin	⇑	Reduced NK effector functions Inhibition of positive intracellular signaling	Lu et al. (68); Martinet et al. (67)
sMIC	NKG2D ligand	⇑	Reduced NK effector functions Downregulation of NK activating receptors	Groh et al. (86); Jinushi et al. (84); Xiao et al. (89)
**Cell bound**				
mMIC	NKG2D ligand	⇓	Resistance to NK cytotoxicity	Jinushi et al. (84)
B7-H6	NKp30 ligand	⇓	Resistance to NK cytotoxicity	Fiegler et al. (104)
HLA class I	KIR/NKG2A ligands	⇑	Resistance to NK cytotoxicity	Carbone et al. (105); Bossard et al. (106); Sarkar et al. (107)
CD200	Membrane glycoprotein	⇑	Reduced NK effector functions Downregulation of NK activating receptors	Moreaux et al. (112); Coles et al. (110)
2B4	Activating receptor	⇓	Reduced NK effector functions	Fauriat et al. (122); Costello et al. (123)
NKG2D	Activating receptor	⇓	Reduced NK effector functions	Fauriat et al. (122); Costello et al. (123)
NCRs	Activating receptors	⇓	Reduced NK effector functions	Fauriat et al. (122); Costello et al. (123)
DNAM-1	Activating receptor	⇓	Reduced NK effector functions	El-Sherbiny et al. (128)
PD-1	Immune checkpoint receptor	⇑	Reduced NK effector functions	Benson et al. (125); Gorgun et al. (126); Beldi-Ferchiou et al. (124)
KLRG1	Co-inhibitory receptor	⇑	Reduced NK effector functions	Ponzetta et al. (127)

*TM, tumor microenvironment; PGE2, prostaglandin E2; sMIC, soluble MIC; mMIC, membrane-bound MIC; KIRs, killer immunoglobulin-like receptors; NCRs, natural cytotoxicity receptors; PD-1, programmed cell death protein 1/programmed cell death protein ligand 1; KLRG1, killer cell lectin-like receptor subfamily G member 1*.

*⇑ denotes increase; ⇓ denotes decrease*.

## Immune Checkpoint Blockade of NK Cells

### Inhibitors of the PD-1/PD-L1 Pathway

Programmed death receptor-1 is a transmembrane protein expressed on the surface of antigen-activated T and B cells. It has two ligands, PD-L1 and PD-L2. PD-L1 is expressed on both antigen-presenting cells/dendritic cells and a wide spectrum of non-hematopoietic cells. PD-1/PD-L1 interactions physiologically counter T cell stimulatory signals and allow T cell homeostasis and self-tolerance by suppressing activation and proliferation of autoreactive T cells. PD-1/PD-L1 binding delivers an inhibitory costimulatory signal that induces a state of T cell exhaustion that prevents activation and proliferation of T cells. Unlike NK cells from healthy donors, NK cells from MM patients express PD-1 (Figure 1A), suggesting that NK cells from healthy donors do not express PD-1 (Figure 1A), however, NK cells from MM patients do. This may show that a functional change in NK cells in response to MM may cause an immunosuppressive microenvironment for MM to grow. In the light of these observations and the broad expression of PD-1 and its ligands in the MM microenvironment, the PD-1/PD-L1 pathway may play a pivotal role in the immune evasion of MM cells (Figure 1B).

A role for the PD-1/PD-L1 signaling pathway in the NK cell immunoresponse against MM and of the anti-PD1 antibody CT-011 was first shown by Benson et al. (125). CT-011 was demonstrated to enhance human NK cell function against autologous, primary MM cells by affecting NK cell trafficking, immune complex formation with MM cells, and cytotoxicity toward MM cells expressing PD-L1 while sparing normal cells (Figure 1B).

It was also shown that lenalidomide had the ability to down regulate PD-L1 on primary MM cells and, by so doing, increase NK cell functions against MM. Thus, targeting the PD-1/PD-L1 pathway may become a feasible clinical strategy in MM, especially in patients with persistent residual disease (131).

One preliminary phase I study reported on 17 patients treated with pembrolizumab, a PD-1 inhibitor, in combination with lenalidomide and dexamethasone (132). Overall response and very good partial response rates were 76 and 23%, respectively. Some 75% of patients achieved stable disease. Many patients were heavily pretreated with other lines of therapy. Almost all patients, however, experienced at least one adverse event with anemia, neutropenia, thrombocytopenia, fatigue, hyperglycemia, and muscle spasms being the most common. Two other recent studies with nivolumab showed acceptable toxicity but no objective responses (133, 134). Efficacy assessment of nivolumab, alone or in combination, is ongoing.

More recently, a novel subpopulation of human NK cells expressing high levels of PD-1 have been identified in ovarian cancer, characterized by low proliferative responses, and impaired antitumor activity that can be partially restored by antibody-mediated disruption of PD-1/PD-L1 interaction (135).

Future studies to evaluate the real therapeutic role of anti-PD-1 antibodies, maybe in combination with other agents with potent anti-myeloma activity such as lenalidomide, are warranted.

### KIR-Specific Immune Checkpoint Inhibition

The role of NK cells as graft-vs.-myeloma effectors was first investigated in preclinical models. Frohn et al. described for the first time the killing ability of NK cells against three different MM cell lines. The mean NK cell killing ability on MM samples ranged from 23 to 34.5% (136). Moreover, KIR-ligand mismatch in T cell-depleted allogeneic stem cell transplantation reduced the relapse incidence in MM recipients. The impact of KIR-ligand mismatch was assessed in a cohort of 73 MM patients who received reduced-intensity unrelated donor transplants. KIR-ligand mismatch in the graft-vs.-host disease direction was significantly associated with lower risk of relapse (HR: 0; *p* < 0.0001) (137).

**Figure 2:**
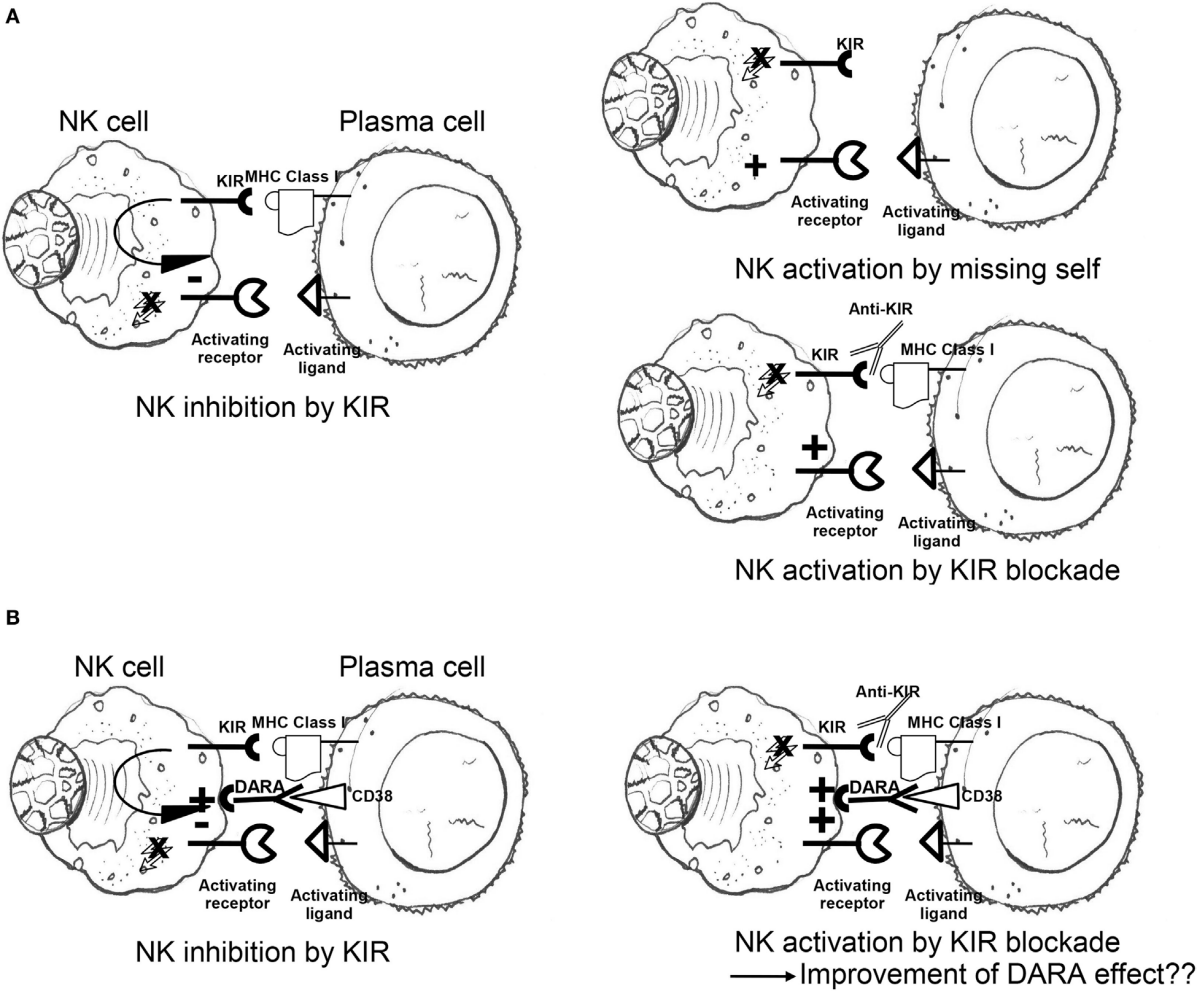
**(A)** Engagement of self-MHC class I by inhibitory KIR results in dominant-negative signals blocking competing activation responses; lack of MHC class I molecules triggers NK cell killing (missing-self recognition); inhibitory KIR blockade by anti-KIR mAbs abrogates KIR-mediated inhibition regardless of MHC class I ligand expression on target surface (“induced” missing self). **(B)** Negative signals transduced by inhibitory KIR antagonize anti-CD38 (DARA)-induced antibody-dependent cellular cytotoxicity, potentially dampening NK cytotoxicity to plasma cells; addition of KIR checkpoint inhibitors may potentiate the positive effects of DARA on NK cytotoxicity of malignant plasma cells (see also main text). NK, natural killer; KIR, killer cell immunoglobulin-like receptor; MHC, major histocompatibility complex; DARA, daratumumab.

To exploit this pathway, Romagné et al. generated an IgG monoclonal antibody, 1-7F9, against three different KIRs (KIR2DL-1, KIR2DL-2, and KIR2DL-3) to enhance the NK cells antitumor effect. This checkpoint inhibitor augmented NK cell-mediated lysis of HLA-C-expressing tumor cells without interfering with normal peripheral blood (PB) mononuclear cells (138) (Figure 2A). The therapeutic potential of 1-7F9 was then demonstrated in preclinical mouse models, providing the platform for translational studies in humans (139).

The drug IPH2101, formerly 1-7F9, was tested in a phase I trial in 32 patients with relapsed/refractory MM. IPH2101 was administered for up to four 28-day cycles, in 7 dose-escalated cohorts (0.0003–3 mg/kg). Only one patient developed severe toxicity, characterized by grade 4 acute renal failure with hyperkalemia and hyperuricemia. From a biological point of view, the drug determined the full saturation of NK inhibitory KIRs (140). Furthermore, lenalidomide and IPH2101 were investigated as a novel, steroid-sparing, dual immunotherapy in 15 MM patients: the biological endpoint of full KIR occupancy was achieved, 5 patients had a response, and 5 severe adverse events were reported (141).

In an open-label, single arm two-stage phase II trial, IPH2101 was employed at the dose of 1 mg/kg every other month for six cycles in nine patients with smoldering MM. Despite the promising results from preclinical and phase I studies, the trial was terminated before planned second stage due to lack of patients meeting the primary objective (50% decline in M-protein) (142).

A recombinant version of IPH2101 was developed with a stabilized hinge (lirilumab). A phase I study of the safety and tolerability of lirilumab with elotuzumab in myeloma patients is currently in progress. Of note, lirilumab recognizes both the inhibitory KIR2DL1, -L2, and -L3 and the activating KIR2DS1-2. Therefore, lirilumab-mediated modulation of intracellular signals is expected to vary according to patient’s HLA class I genetic background and KIR receptor repertoire.

*In vitro* experiments showed that KIR2D molecules are removed from NK cells surface by trogocytosis. This phenomenon culminated in a strong reduction of NK cell cytotoxic function correlating with the loss of free KIR2D surface molecules (143). These data favor future protocol designs where lirilumab is administered in combination with other NK cell-activating agents, rather than as single agent.

## Immunomodulatory Drugs and Monoclonal Antibodies

### Lenalidomide

Lenalidomide, a thalidomide analog, is an immunomodulatory drug with multiple mechanisms of action in MM. It is currently approved in both EU and USA in association with dexamethasone for the maintenance treatment of patients with newly diagnosed MM who have undergone an autograft. Four pivotal phase III studies have associated lenalidomide with improved progression-free survival and better overall response rates (144–147). Although lenalidomide has also been associated with increased risk of a second primary cancer, the overall survival benefits outweigh the risk (148).

Due to failure of single-agent anti-KIRs in phase II studies, researchers from multiple institutions investigated possible combined therapies. *In vitro*, the immunomodulatory agent lenalidomide was responsible of NK cell expansion and activation associated with malignant cells apoptosis (149). On this platform, Benson et al. tested the cytotoxicity of IPH2101 in combination to lenalidomide against MM cell lines U266 and K562 (139, 140). Healthy donor NK cells pretreated with lenalidomide or IPH2101 alone and combined showed increased IFN-γ production against primary MM cells compared to controls (*p* < 0.05). Furthermore, NK cells pretreated with both lenalidomide and IPH2101 led to the highest IFN-γ peak. The statistical interaction of *p*-value was 0.0182, suggesting a synergistic effect between the two drugs. Then, healthy donor PB mononuclear cells (PBMCs) incubated as control or with lenalidomide and/or with IPH2101 were used as effectors against U266 MM cell targets. Lenalidomide increased the specific release, a surrogate for cytotoxicity, by around 1.39-fold relative to control (*p* < 0.01). IPH2101 increased the specific release by 1.48-fold (*p* < 0.01). The two drugs combined increased the specific release by 2.09-fold relative to control (*p* < 0.001), which means a significantly higher cytotoxic effect than either lenalidomide or IPH2101 alone. Patient-derived NK cell cytotoxicity against autologous MM targets was enhanced by the combination of lenalidomide plus IPH2101 (128 ± 9 spots/well) compared with control conditions (81 ± 7 spots/well). Based on *in vitro* results, the authors evaluated the efficacy of the anti-KIR 5E6 in lenalidomide pretreated mice. The tumor burden was significantly reduced when the combination of 5E6 and lenalidomide was employed, in comparison to controls (*p* < 0.005). These data provide the basis for the translation of IPH2101 and lenalidomide combination in phase I and II studies.

### Daratumumab (DARA)

Daratumumab is an IgGk monoclonal antibody targeting CD38, a cell surface protein that is overexpressed on MM cells (150, 151). Preclinical studies have shown that DARA induces MM cell death through several mechanisms, including complement-dependent cytotoxicity (152), ADCC (153), antibody-dependent cellular phagocytosis (154), and apoptosis (155). The drug showed efficacy as single agent in heavily pretreated MM patients or in combination with bortezomib and dexamethasone (156). When combined to lenalidomide, the DARA cell-mediated MM cell clearance was enhanced due to lenalidomide-dependent NK cell activation. In the light of preclinical results of lenalidomide in combination with anti-KIR agents, Nijhof et al. hypothesized that the NK cell-mediated cytotoxicity induced by DARA could be enhanced by anti-KIRs (Figure 2B). The effect could be further improved through the association with lenalidomide which stimulates the proliferation of NK cells and activates them (157), overcoming NK cells depletion induced by DARA itself (158).

### Elotuzumab

**Figure 3:**
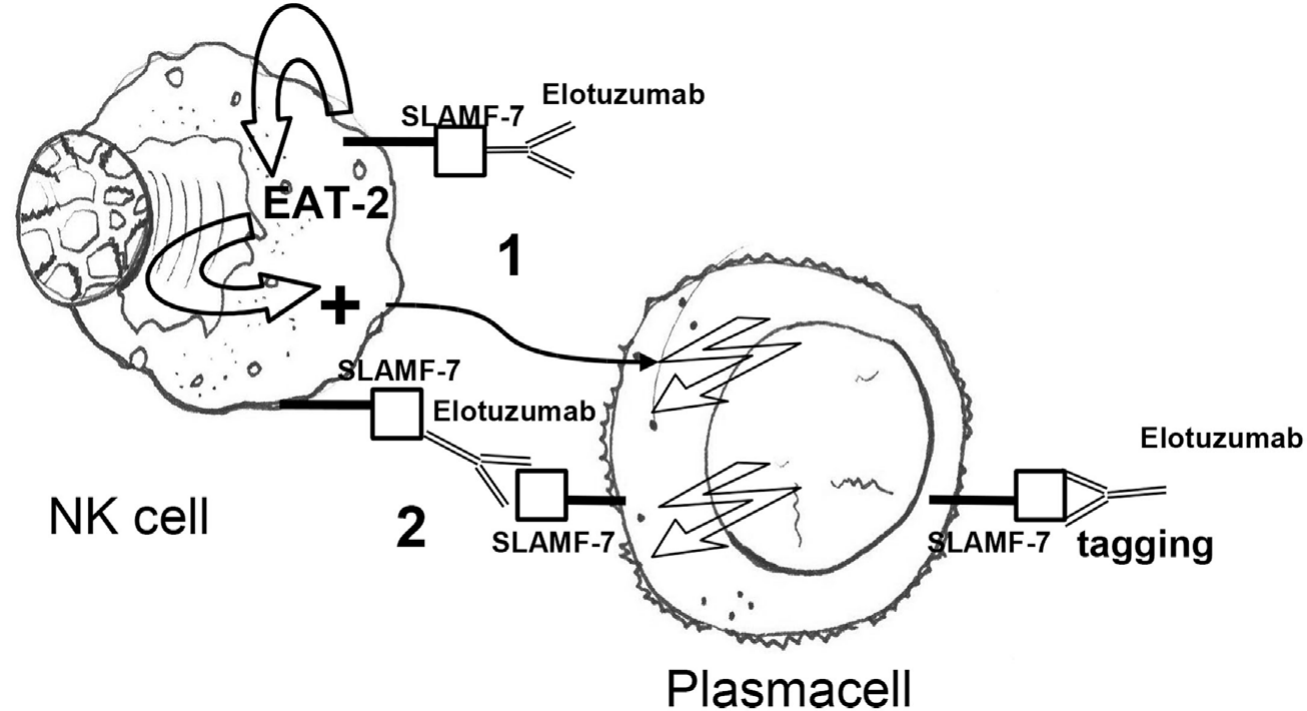
Elotuzumab activates NK cells *via* (1) an indirect mechanism, i.e., binding of the extracellular portion of SLAMF7 and recruitment of the EAT-2 adaptor protein and (2) a direct mechanism, i.e., antibody-dependent cellular cytotoxicity in response to SLAMF7 tagging on plasma cells. Owing to the absence of EAT-2 in plasma cells, elotuzumab engagement does not cause activation of plasma cells. NK, natural killer; EAT-2 Ewing’s sarcoma-associated transcript 2; Fc, fragment crystallizable; Fab, fragment antigen binding.

Initially, Hsi et al. described a humanized antibody, HuLuc63, which specifically targeted CS1 (CCND3 subset 1, CRACC, and SLAMF7), a cell surface glycoprotein that had not previously been associated with MM cells. By flow-cytometry, HuLuc63 showed specific staining of CD138^+^ myeloma cells, NK cells, NK-like T cells, and CD8^+^ T cells. HuLuc63 showed significant *in vitro* ADCC against primary myeloma cells as targets and allogeneic or autologous NK cells as effectors. The authors concluded that HuLuc63 could eliminate MM partly through NK-mediated ADCC and targeting CS1 with HuLuc63 could become a novel treatment strategy (159). Tai et al. also showed that HuLuc63 was effective in inducing ADCC against primary MM cells resistant to novel therapies such as bortezomib and HSP90 inhibitor. Moreover, pre-treatment with conventional or novel anti-MM agents enhanced HuLuc63-induced MM cell lysis (160). Collins et al. also hypothesized that elotuzumab may have other mechanisms of action. A number of findings clearly suggested that elotuzumab may enhance NK cell function beyond ADCC. Elotuzumab was shown to induce NK cell activation by binding to CS1 which promotes cytotoxicity against CS1^+^ MM cells but not against autologous CS1^+^ NK cells. Moreover, NK cell activation was shown to be dependent on differential expression of the signaling intermediary EAT-2 which is present in NK cells but absent in primary, human MM cells (161). Therefore, HuLu63 enhances NK cell cytotoxicity to MM *via* a dual mechanism (Figure 3). The synergy between current anti-CS1 antibody elotuzumab, formerly known as HuLuc63, and bortezomib was also shown by van Rhee et al. (162). Elotuzumab was approved by FDA in 2015 for the treatment of MM, specific for signaling lymphocytic activation molecule-F7 (SLAMF7, or CS1) (163). As previously mentioned, SLAMF7 is a member of the Ig gene superfamily, almost universally expressed (>95%) on the surface of marrow MM cells, but not on normal tissues, with restricted expression on specific lymphocytes including NK cells. SLAMF7 determines activating or inhibitory effects on NK cells depending on the expression or not of EAT-2, an adapter protein (Figure 3). Given that MM cells lack EAT-2, the molecular mechanism by which SLAMF7 mediates inhibition in NK cells was investigated by Guo et al. It was shown that the inhibitory effects of SLAMF7 in EAT-2^−^ NK cells was mediated by a mechanism implicating lipid phosphatase SHIP-1, Src kinases, and protein tyrosine phosphatase CD45. Coupling of SLAMF7 to SHIP-1 was highly compromised in MM cells. This correlated with a lack of CD45, which is required to activate Src family kinases in hematopoietic cells and was needed to initiate SLAMF7 inhibitory signals. This defect may explain why elotuzumab eliminates MM cells by an indirect mechanism that involves NK cells activation (164, 165). An elegant preclinical model clearly showed that elotuzumab activates NK cells and promotes myeloma cell death in healthy donor PB lymphocyte (PBL)/myeloma cell cocultures (166). Moreover, the combination of elotuzumab plus lenalidomide demonstrated higher anti-myeloma activity on established *in vivo* MM xenografts and in *in vitro* PBL/myeloma cell cocultures than either agent alone. In the same study, it was interestingly shown that the increased NK cell anti-myeloma functions were also due to increased secretion of IL-2 and production of TNF-α that combined to enhance NK cell activation and MM cell killing. All these findings supported the clinical application of combination strategies. Elotuzumab initially showed activity in combination with lenalidomide and dexamethasone in a phase I and a phase Ib-2 clinical studies in relapsed/refractory MM (167, 168). In a subsequet randomized study, patients with relapsed/refractory MM received either elotuzumab with lenalidomide and dexamethasone, or lenalidomide and dexamethasone alone. Patients who received a combination of elotuzumab, lenalidomide, and dexamethasone had a significant relative reduction of 30% in the risk of disease progression or death (169). Finally, Jakubowiak et al. reported on a phase II study in relapsed/refractory MM patients where combined elotuzumab/bortezomib/dexamethasone were compared with bortezomib/dexamethasone until progression or unacceptable toxicity. Overall, elotuzumab appeared to provide clinical benefit without clinically significant toxicity when combined with bortezomib (170).

### IDO Inhibitors

Indoleamine 2,3-dioxygenase plays a pivotal role in the metabolic cascade that converts the essential amino acid l-tryptophan (Trp) into l-kynurenine (Kyn). Moreover, IDO has also been shown to be involved in the establishment and maintenance of peripheral tolerance. This function may partly be due to IDO1 capacity to restrict the microenvironmental availability of Trp and to increase the accumulation of Kyn and derivatives. The expression of IDO on neoplastic lesions may help cancer to escape immunosurveillance. IDO inhibitors (i.e., 1-methyltryptophan) have therefore become a new class of anti-cancer agents. Current models imply that IDO limits both innate and adaptive immunoresponses by depleting immunoeffector cells of Trp (171, 172) and by promoting the accumulation of Kyn and its derivatives 3-dydroxykynurenine and 3-hydroxyanthranilic acid (173, 174). These derivatives have been shown not only to exert cytostatic and cytotoxic effects on several immunoeffectors including CD8 T cells, NK cells, and invariant NKT cells (173–175) but also to inhibit TH17 cells and to promote the differentiation of naıve CD4 T cells into T_regs_ and tolerogenic activity of dendritic cells (174–181). Interestingly, Bonanno et al. investigated IDO expression in 25 symptomatic MM and in 7 with either MGUS or smoldering MM (182). IDO-driven tryptophan breakdown was correlated with the release of hepatocyte growth factor (HGF) and with the frequency of T_reg_ cells and NY-ESO-1-specific CD8 T cells. Kyn was increased in 75% of MM patients and correlated with the expansion of T_reg_ cells and the contraction of NY-ESO-1-specific CD8 T cells. *In vitro*, primary MM cells promoted the differentiation of allogeneic CD4 T cells into T_reg_ cells and suppressed IFN-γ/IL-2 secretion while preserving IL-4 and IL-10 production. Both T_reg_ expansion and inhibition of Th1 differentiation were partly reverted by d,l-1-methyl-tryptophan, an inhibitor of IDO. Of note, HGF levels were higher within the marrow microenvironment of patients with IDO^(+)^ MM as compared with patients with IDO^(−)^ MM. The antagonism of MET receptor for HGF with SU11274, a MET inhibitor, prevented HGF-induced AKT phosphorylation and resulted in reduced IDO protein levels and functional activity. These findings suggest that IDO expression may contribute to immunosuppression in patients with MM. IDO inhibitors are currently being tested either as single agent or in combination with other anti-cancer drugs in a number of solid tumors even though this class has not yet been evaluated in MM.

## NK Cells and Their Role in Allografting: Lessons from Acute Leukemias

Some of the most convincing proofs of the potential of NK cells as immunotherapeutic tools derive from evidences accumulated over the past two decades in the setting of allogeneic hematopoietic stem cell transplantation (allo-HSCT). The interest in NK cell immunobiology stemmed mainly from two observations: (a) NK cells are the first lymphocyte subset to recover after transplantation, often reaching percentages and absolute counts superior to those commonly observed in healthy subjects (183, 184), and (b) since KIRs and HLA ligands are encoded on different, independently inherited, chromosomes (chromosome 19 and 6, respectively), the KIR gene repertoire transferred from the donor into the host is often different posing the issue whether, and how, an efficient NK cell functionality can be achieved (185–187).

Two hallmark studies came from the Stanford group (188, 189) where it was demonstrated that after a variable number of months following HLA-matched, KIR-mismatched allo-HSCT, the NK cell repertoire is dominated by lymphocytes displaying an immature phenotype (CD56^bright^ and CD94/NKG2A^+^) and then it stabilizes and becomes similar to that of donor origin. Moreover, these studies highlighted significant differences in the repertoire recovery kinetics among patients, and clearly correlated impaired NK cell reconstitution with occurrence of post-transplant complications. The development of a HSCT platform which allowed to safely infuse HLA-haploidentical grafts set the stage for some of the most exciting discoveries in transplant biology and translational potential of NK cells.

Starting from preclinical studies on the tolerogenic potential of “stem cell megadoses” (190), the Perugia team developed a protocol which combined a highly immunosuppressive myeloablative conditioning regimen with the infusion of high doses of extensively T cell-depleted HLA-haploidentical hematopoietic stem cells (HSCs). Full donor engraftment of the partially incompatible HSCs was successfully achieved, and despite the absence of post-transplant pharmacological prophylaxis, neither acute nor chronic GvHD ensued (191). This elegant and technologically advanced HSCT platform offered the opportunity to investigate the metrics of NK cell reconstitution in a partially HLA-mismatched host and in the absence of confounding factors such as alloreactive T cells or immunosuppressive drugs. This highly favorable environment further boosted the early expansion of NK cells that had been already described in other transplant settings, and, importantly, led to the appearance of donor-derived NK cells with alloreactivity against the host (192).

Velardi et al. brilliantly described the principles by which NK cell alloreactivity developed and designed an algorithm to easily predict it. Based on this model—later defined as “ligand-ligand” or “KIR-ligand mismatch” model—post-transplant alloreactivity is unleashed when the donor carried one or more KIR ligands (i.e., HLA class I alleles encompassing the Bw4, C1, or C2 motifs) absent in the host. In this setting, inhibitory KIRs expressed on the surface of donor-derived NK cells—which, in the host, are continuously engaged by their respective ligands in the pre-transplant phase—do not find their cognate HLA molecules on host cells and tissues leading to a perception of “missing self” that activates an alloreactive response (193, 194).

One of the most striking observations by the Perugia group was that NK alloreactivity did not result in clinical GvHD, but, conversely, led to a potentially eradicating mechanism of residual leukemic cells reducing relapse incidence and risk of graft failure and GvHD (195, 196). Over the following years, several studies confirmed and consolidated the evidence that in T cell-depleted haploidentical HSCT NK cell alloreactivity represents the main driver of the graft-vs.-leukemia effect and a major predictor of overall clinical outcomes in both adults (197–199) and children (200, 201). In addition, these studies were a major drive for the development of cell therapy protocols in which haploidentical KIR ligand-mismatched NK cells were infused in leukemic patients after lymphodepleting chemotherapy with highly promising results (202–204).

In more recent years, several new platforms of haploidentical HSCT have been developed, mainly with the aim at improving T cell immune reconstitution and at reducing post-transplant infectious complications (205). In this “new era,” studies on the impact of KIR-ligand mismatches on transplant outcomes reported some conflicting results. For instance, it was shown that infusion of unmanipulated BM grafts or of donor post-transplant T cell add-backs may mask, or blunt, the effectiveness of NK cell alloreactivity (206–208). By contrast, other recent haploidentical HSCT platforms based on the selective depletion of αβ T cells or on the infusion of balanced doses of conventional and regulatory T cells appeared to better preserve the positive effect of KIR ligand mismatches (209, 210).

In partially HLA-mismatched unrelated donor HSCT, either from adult volunteers or cord blood (CB) units, the potential role of NK alloreactivity has also been a matter of debate. Some studies supported a positive role of KIR ligand mismatches (211–213) and others found no significant advantage or even adverse effects (214–216).

To overcome these inconsistencies, several alternative immunogenetic models have been developed to better predict NK cell-driven effects on transplant outcomes. In particular, Cooley et al. focused on the donor genetic repertoire and demonstrated in a number of independent studies that donors with a KIR gene asset enriched in activating receptors—group B KIR haplotypes—can provide a superior relapse-free survival after unrelated HSCT for leukemias (217–219). Another model which takes into account both donor activating KIR asset and donor/recipient HLA typing has been proposed and validated by Venstrom et al. In an analysis on more than 1,200 unrelated HSCTs, the authors observed that the presence of donor-activating receptor KIR2DS1 and of HLA-C1 ligands provided a significant protection from relapse, further enhanced in case of recipient HLA-C1 positivity (220).

Despite the multiplicity of models proposed over the years in the setting of allo-HSCT, not all the immunogenetic mechanisms that regulate NK cell interactions and alloreactivity have fully been understood. However, it is widely assumed that NK cell alloreactivity is instrumental in control and eradication of hematological malignancies.

## NK Cell Therapies

### Expanded NK Cells for MM Treatment

Expansion of NK cells from PBMC of patients with MM has been achieved using a culture system supplemented with IL-2 and OKT3 (221). NK cells could be extensively propagated (average 1,625-fold expansion in 20 days) and displayed increased levels of activating receptors as well as cytotoxicity to the NK-susceptible K562 line and to autologous MM cells (222). Another NK cell expansion strategy for MM immunotherapy is based on the artificial feeder K562 transfected with CD137L and membrane-bound IL-15. This technique allowed extensive *in vitro* NK cell propagation (average 447-fold, range 20–10,430 on harvest day, i.e., days 10–14). Transfer of these cells into a xenogeneic model of high-risk MM resulted in myeloma growth inhibition and protection against osteolysis (223). The same group tested the safety, persistence, and activity of expanded NK cells in seven heavily pretreated patients with high-risk relapsed myeloma: no serious adverse events related to NK cell infusion was observed. Moreover, the infusion of fresh, rather than cryopreserved, cells resulted of fundamental importance for their *in vivo* expansion. Two/seven patients showed some responses which lasted for at least 6 months (224). More recently, human studies were performed with allogeneic, KIR ligand-mismatched NK cells from haploidentical family donors. NK cells were cytotoxic to K562, the myeloma line U266, and recipient primary MM cells. Fifty percent of the patients with advanced MM achieved near complete remission when these cells were infused prior to autologous SCT (225). Another phase I clinical trial (NCT02481934) evaluated safety and efficacy of multiple infusions of activated and expanded NK cells in combination with lenalidomide- or bortezomib-based regimens (226). Five heavily pretreated refractory/relapsed patients were enrolled. NK cells were activated and expanded for 3 weeks with K562mb15-41BBL cells. Patients received four cycles of new drug-based treatment with two infusions of 7.5 × 10^6^/kg NK cells. Four patients showed stable disease while on NK cell treatment, two showed a 50% reduction in BM plasma cell infiltration and one obtained a response >1 year. No major toxicities were reported. Expanded NK cells showed a highly cytotoxic phenotype and *in vitro* killing and were detected in both BM and PB of treated patients. While efficacy and safety of multiple NK cell infusions need further assessment, these data suggest that repeated transfer of *in vitro* activated and expanded NK cells into MM patients is feasible and may result in clinical benefit when combined with anti-myeloma drugs.

### CB NK Cells

Umbilical CB represents a promising source of allogeneic NK cells. However, GMP-grade large scale *ex vivo* expansion is indispensable to generate CB-derived NK (CB-NK) cell doses that may be used in the clinical setting. Shah et al. recently described a strategy for the expansion of NK cells from cryopreserved CB units (227). By co-culturing for 14 days CB units using artificial antigen-presenting feeder cells (aAPC), a highly expanded cell product (average 1,848- and 2,389-fold in 14 days from fresh and cryopreserved samples, respectively) of 95% purity for CB-NK cells and less than 1% CD3^+^ cells was obtained. Despite differences in the expression of certain cytotoxicity receptors, aAPC-expanded CB-NK cells were phenotypically very similar to CB-NK cells expanded with IL-2 alone. Most importantly, aAPC-expanded CB-NK cells clearly showed cytotoxicity against both *in vitro* MM targets and *in vivo* anti-myeloma activity in a xenogenic mouse model. The same group investigated the mechanisms of CB-NK-mediated cytotoxicity against MM cells (228). Interestingly, a mechanism of transmissible cell death between cells induced by lipid–protein vesicles transferred from CB-NK to MM cells was described. Moreover, these vesicles were capable of migrating from recipient MM cells to neighboring MM cells enhancing cytotoxicity of CB-NK. Altogether, these findings supported the development of CB-NK-based cellular therapies for the treatment of MM. An encouraging first-in-human study of CB-NK cells for MM patients undergoing high dose chemotherapy and autologous transplantation was recently conducted (229). Patients received lenalidomide at a dose of 10 mg from day −8 through −2, standard melphalan at 200 mg/m^2^ on day −7. CB-NK cells were infused on day −5 and the autograft performed on day 0. Twelve patients were treated with different dose levels. Most patients were heavily pretreated and had high-risk cytogenetics. Overall CB-NK cells with an activated phenotype (NKG2D^+^/NKp30^+^) were detected *in vivo* in six patients. Importantly, no signs/symptoms of GVHD were observed. Eight patients achieved at least near complete remission and two additional patients a very good partial response. After a median follow-up of 21 months, four patients relapsed or experienced progressive disease.

### CAR-NK for Myeloma

The impressive clinical results obtained in patients with B cell malignancies with the infusion of T cells genetically modified to express synthetic chimeric antigen receptors (CARs) against the lineage-specific surface antigen CD19 represented a turning point in the history of cancer immunotherapy (230–236) Intriguingly, T cells engineered with an anti-CD19 CAR were capable to induce complete remission also in a patient with MM. However, given that the large majority of malignant plasma cells do not express CD19, studies to understand the mechanism that underlie this unexpected observation are currently in progress (237). More recently, a number of CARs have been developed to specifically target surface antigens expressed by pathological plasma cells, including CD38 (238, 239), CD138 (240), B cell maturation antigen (241, 242), κ light chains (243), SLAMF7 (244), and CD44v6 (245). However, despite their tremendous efficacy, CAR-T cells have also raised concerns on their short- and long-term toxicities, in particular the development of life-threatening cytokine release syndrome and the risks of prolonged aplasia of the healthy counterparts of the target tumor—“off tumor/on target toxicity”—and in case of allogeneic CAR-T cells the development of GvHD (246, 247).

To address these issues, genetic modifications with CARs of cells belonging to the innate immune system, and of NK cells in particular, may yield several potential advantages. For instance, most innate cells recognize and eliminate tumors by stereotyped patterns and have been infused into allogeneic recipient without excessive toxicities and with some promising intrinsic antitumor efficacy. Moreover, the short-lived persistence of innate immunocells in an allogeneic host, considered up to now one of the major limitations, may become an added value in case of CARs targeting antigens that are shared with mature cell types for which prolonged aplasia may be a concern (i.e., memory B cells, monocytes, or plasma cells) (248, 249).

Genetic modification of the human NK cell lines NKL and NK-92 by means of a lentiviral vector encoding for anti-SLAMF7 and anti-CD138 CARs has proven feasible. This did not substantially modify the expression profile of transduced cells and conferred selectivity for the target and the ability to kill human malignant plasma cells both in *ex vivo* and in an orthotopic xenograft models (250, 251). Overall, several steps to optimize and validate CAR-modified NK cells should be taken before their possible clinical use. In particular, the choice of the most appropriate NK cell source to be modified is a matter of intense debate (252). Whether freshly isolated NK cells may represent the most physiological choice to achieve sufficient cell doses and transduction efficiency remains unknown. Conversely, NK cells expanded from PB or from progenitor cells may be more easily modified even though their expression profile and functional competence may be negatively affected by prolonged *ex vivo* culture. Finally, immortalized human NK cell lines, such as NK-92, can be very efficiently transduced and expanded in desired numbers even for “off-the-shelf” use even though their cell surface expression of activating receptors is lower than in freshly isolated or expanded NK cells. Moreover, the need to irradiate the cell product before infusion would further limit their *in vivo* persistence (252, 253). A new modality that exploits the combination of the anti-CR38 monoclonal antibody DARA with CD38^(−)^ NK cells armed with CS1 CAR has very recently been described by Wang et al. to treat relapsed MM (254). Given that both CS1 and CD38 are MM-associated antigens, their simultaneous targeting may prevent progression. The same authors previously showed that DARA induces apoptosis in CD38^(+)^ NK cells but not in CD38^(−)^ NK cells. It was then hypothesized that DARA in combination with CD38^(−)^ CS1-CAR NK cells may show a synergistic effect and possibly lead to MM eradication. Long-term follow-up of clinical outcomes of this study are eagerly awaited.

## Future Perspectives

The potent crosstalk between malignant plasma cells and their BM microenvironment plays a central role in MM progression and resistance to current therapies. Novel forms of immunotherapy against MM represent a rapidly developing area in cancer therapy. They include treatment strategies that may be delivered either alone or in combination with currently employed therapy lines such as IMiDs and proteasome inhibitors as well as newer agents (Table 2). Moreover, immunotherapy may attenuate the systemic toxicity of cytotoxic chemotherapy. A robust body of evidence has clearly shown that enhancing host anti-myeloma immunity within the BM microenvironment may lead to a more efficient disease control. NK cells play a pivotal role in the intricate network of cells and signaling pathways that may prevent immune escape mechanisms. NK cells were clearly shown to have potent *in vivo* antileukemia activity in patients undergoing allografting. Recent observations on NK cell functions in MM have become promising immunotherapeutic strategies. New avenues of research have included expansion of NK cells from PB as well as CB, and the generations of specific CAR-NK cells against myeloma-specific antigens. Moreover, MM NK cells express PD-1 whereas NK cells from healthy individuals do not. This phenotypic characteristic may indicate that immunocheckpoint blockade of NK cells may be an area to fully explore given the remarkable results obtained with anti-PD 1 inhibitors in cancer treatment. Altogether, the studies reported in this review show that NK cells hold promise in changing the natural course of MM and that may help restore immunity to MM and thereby improve survival outcomes.

**Table 2:** Summary of current treatments with novel agents for multiple myeloma (MM) potentially affecting natural killer (NK) cell activity.

Agent	Mechanism of action on NK cells	Clinical trials	Reference
PD-1/PD-L1 checkpoint inhibitors	*Block of the recognition of PD-L1 by PD-1 on NK cells* PD1 blockade may neutralize competitive negative signals resulting in enhanced trafficking, immune complex formation, and cytotoxicity of NK cells (Figures 1A,B)	Phase I trial of pembrolizumab with lenalidomide and dexamethasone. Two Phase I trials involving nivolumab showed acceptable tolerability. Efficacy assessment of nivolumab, alone or in combination is ongoing.	Benson et al. (125); San Miguel et al. (132); Suen et al. (133); Lesokhin et al. (134)
HLA-KIR checkpoint inhibitors	*Prevent inhibitory KIR recognition of cognate HLA class I ligands* Blockade of KIR-HLA interactions may neutralize negative signals transduced by inhibitory KIR2DL1/2/3 (Figure 2A)	Anti-KIR monoclonal antibody IPH2101 (1-7F9) determined the full saturation of NK inhibitory KIR in a phase I trial enrolling patients with RR MM. Full KIR occupancy was also achieved in a study combining lenalidomide and IPH2101. In this study, 5 (33%) patients had a response. In a single arm two-stage phase II trial, IPH2101 was employed in 9 patients with smoldering MM. The study was stopped before planned second stage due to lack of patients meeting the primary objective (50% decline in M-protein). A phase I study combining elotuzumab with lirilumab, a recombinant version of IPH2101, is currently in progress	Frohn et al. (136); Benson et al. (139); Benson et al. (140); Benson et al. (141); Korde et al. (142); Carlsten et al. (143)
Daratumumab (DARA)	*ADCC to CD38^+^ MM cells* Cytolytic activity to MM cells triggered by CD16 signaling upon recognition of antibody tagged to CD38 antigen. NK cell-mediated cytotoxicity induced by DARA could be enhanced by lenalidomide and KIR blockade. Other mechanisms: complement-dependent cytotoxicity, antibody-dependent cellular phagocytosis, and apoptosis (Figure 2B)	DARA was tested in combination with bortezomib and dexamethasone in RRMM. The primary end point was progression-free survival. DARA in combination with bortezomib and dexamethasone resulted in a significantly longer progression-free survival than bortezomib and dexamethasone alone	Palumbo et al. (156)
Elotuzumab	*Direct effect: ADCC to MM cells expressing SLAMF7* Indirect effect: activation of SLAMF7^+^ NK cells Dual mechanism of action: (1) NK cell activation *via* SLAMF7 binding and recruitment of the EAT-2 adaptor proteins; (2) NK-mediated ADCC to SLAMF7^+^ MM cells (Figure 3)	Elotuzumab showed activity in combination with lenalidomide and dexamethasone in a phase I and a phase IIb-II clinical studies in RRMM. In a phase III study, patients with RRMM patients were treated with either elotuzumab with lenalidomide and dexamethasone, or lenalidomide and dexamethasone alone. Patients treated with the combination of elotuzumab, lenalidomide, and dexamethasone had a significantly reduced risk of disease progression or death. In a phase II study in RRMM patients, elotuzumab showed clinical benefit without significant toxicity when combined with bortezomib	Lonial et al. (167); Lonial et al. (169); Jakubowiak et al. (170)
IDO inhibitors	*Inhibition of l-tryptophan degradation* Reversal of NK immunosuppression by increased availability of l-tryptophan and reduced accumulation of l-kyreunine	IDO inhibitors are currently used as single agent or in combination in a number of solid tumors. This class has not yet been evaluated in clinical trials in myeloma patients	Uyttenhove et al. (172); Fallarino et al. (173); Bonanno et al. (182)

*PD-1/PD-L1, programmed cell death protein 1/programmed cell death protein ligand 1; KIRs, killer immunoglobulin-like receptors; RR MM, relapsed/refractory MM; ADCC, antibody-dependent cellular cytotoxicity; SLAMF7, signaling lymphocytic activation molecule family 7; IDO, indoleamine 2,3-dioxygenase*.

## Author Contributions

GP and BB contributed to the initial conception and designed of the manuscript. CB, MF, DM, and LG provided study materials and critically reviewed the manuscript. GP, LV, MF, and BB wrote the manuscript. All authors gave the final approval to the manuscript.

## Conflict of Interest Statement

The authors declare that the research was conducted in the absence of any commercial or financial relationships that could be construed as a potential conflict of interest.
